# Peripheral Giant Cell Granuloma of the Posterior Mandible Mimicking Malignancy: A Case Report

**DOI:** 10.7759/cureus.97794

**Published:** 2025-11-25

**Authors:** Kajal Bhindora, Rajshekhar Halli, Manjula Hebbale

**Affiliations:** 1 Oral and Maxillofacial Surgery, Bharati Vidyapeeth (Deemed to be University) Dental College & Hospital, Pune, IND; 2 Oral Medicine and Radiology, Bharati Vidyapeeth (Deemed to be University) Dental College & Hospital, Pune, IND

**Keywords:** gingival lesion, histopathology, malignancy, peripheral giant cell granuloma, posterior mandible

## Abstract

Peripheral giant cell granuloma (PGCG) is a benign, reactive lesion of the soft tissue that arises exclusively from the gingiva or alveolar mucosa, usually secondary to local irritation or trauma. Although it is most commonly found in the anterior region, its occurrence in the posterior mandible is uncommon. We present a case involving a 48-year-old male with a gradually enlarging swelling in the right posterior mandible. Clinical, radiographic, and histopathological evaluations confirmed the diagnosis of PGCG. The lesion was surgically excised under general anesthesia, with satisfactory healing and no recurrence observed at an eight-month follow-up.

## Introduction

Peripheral giant cell granuloma (PGCG) is a benign, reactive soft-tissue lesion arising exclusively from the gingiva or alveolar mucosa. It develops from reactive proliferation of periodontal or periosteal tissues secondary to persistent irritation [1]. Clinically, PGCG presents as a sessile or pedunculated nodular mass that is red to bluish-purple in color and may ulcerate due to masticatory trauma. Although usually painless, it can bleed easily and occasionally interfere with mastication [2].

Epidemiologically, PGCG accounts for approximately 5-10% of all reactive gingival lesions [1]. It may occur at any age but most frequently affects adults in the fourth to sixth decades, with a slight female predominance, possibly related to hormonal influences [3]. The lesion is more common in the mandible than in the maxilla and tends to occur anteriorly rather than posteriorly [3]. Posterior mandibular involvement, as in the present case, is uncommon and may clinically and radiographically simulate more aggressive pathologies [4].

Radiographic examination often shows superficial “cupping” resorption of the alveolar bone caused by pressure from the lesion rather than true invasion [2]. However, large or long-standing lesions may occasionally appear destructive on imaging. A definitive diagnosis relies on histopathological evaluation, which characteristically demonstrates multinucleated giant cells within a fibrovascular stroma containing hemorrhage, hemosiderin, and inflammatory cells [5,6].

Treatment involves complete surgical excision down to the periosteum and removal of local irritants to prevent recurrence, which has been reported in 5-11% of cases [1,3]. Although biologically benign, PGCG may occasionally present with alarming clinical or radiologic features that mimic malignancy.

The present report describes a rare case of PGCG of the posterior mandible in a 48-year-old male. The lesion exhibited destructive features on the CT scan, initially suggesting a malignant process. This case highlights the diagnostic importance of correlating clinical, radiographic, and histopathologic findings to distinguish between reactive lesions and neoplastic counterparts, guiding appropriate management.

## Case presentation

A 48-year-old male presented to the oral and maxillofacial surgery outpatient department with a history of progressively enlarging swelling in the right posterior mandible over two to three months. He reported no symptoms prior to this period but gradually noticed a firm, slow-growing mass on the right side of his lower jaw. The swelling remained painless. His medical and habit histories were unremarkable, and no identifiable precipitating factor, such as trauma, was noted.

**Figure 1:**
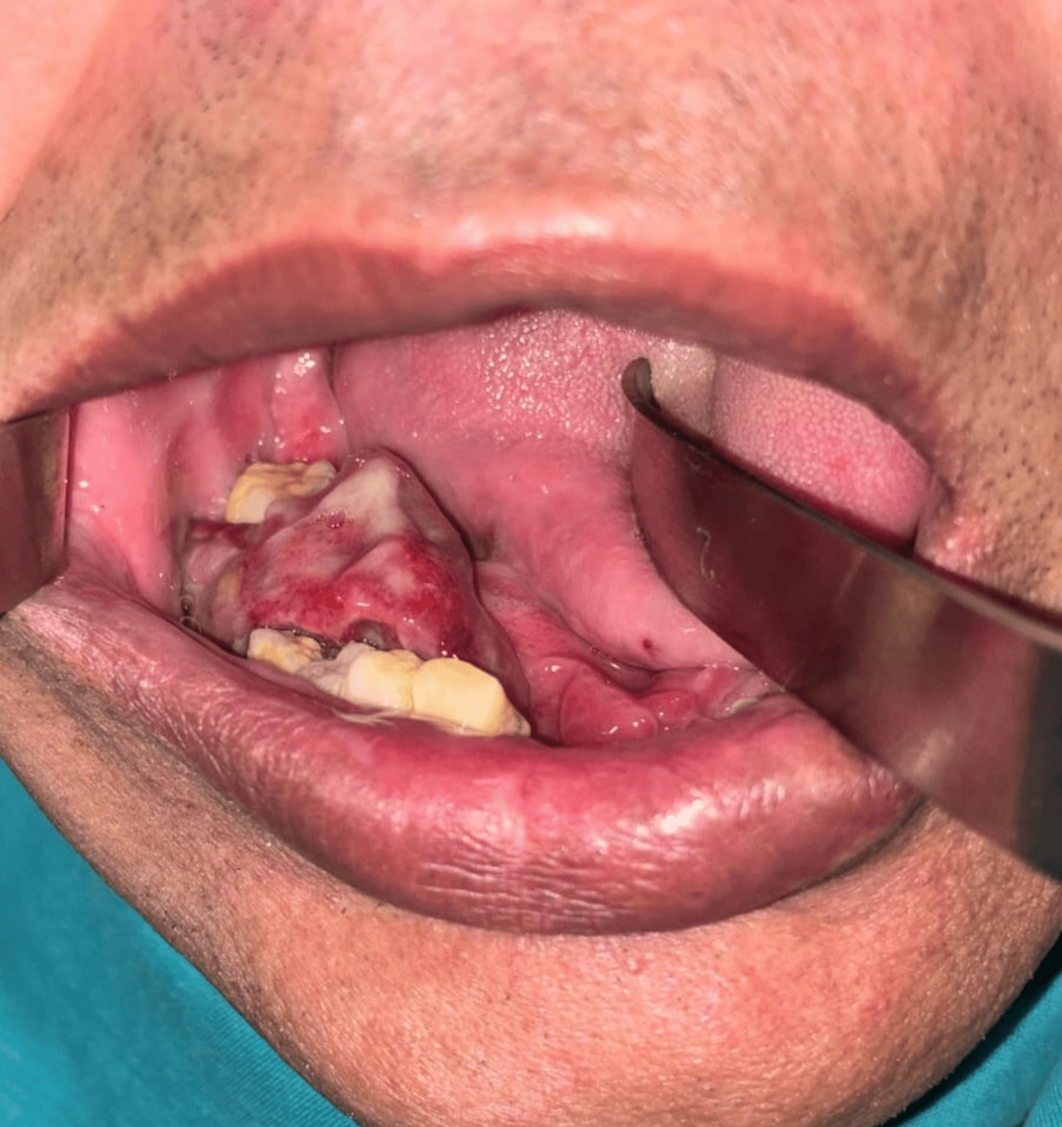
Intraoral photograph showing a well-defined ulceroproliferative growth in the right posterior mandible

Extraoral examination revealed a firm, non-tender swelling in the right submandibular region extending toward the mandibular angle. The overlying skin appeared normal, with no erythema or temperature changes. No cervical lymphadenopathy was observed. Intraorally, a sessile, firm mass was identified distal to tooth 46, extending toward tooth 48 (Figure 1). The lesion occupied the buccal and lingual vestibular regions and measured approximately 2.5 × 1.5 cm. The surface exhibited a reddish-purple coloration with areas of superficial ulceration. The ulceration partially involved the underlying extraction socket, and the margins appeared rolled and indurated. Significant mobility was noted in teeth 45, 46, and 48 (Grade III), while tooth 44 demonstrated Grade II mobility, primarily due to localized alveolar bone destruction. Occlusion was satisfactory, without evidence of traumatic contacts.

An incisional biopsy performed at this stage suggested a giant cell-rich reactive lesion consistent with PGCG.

**Figure 2:**
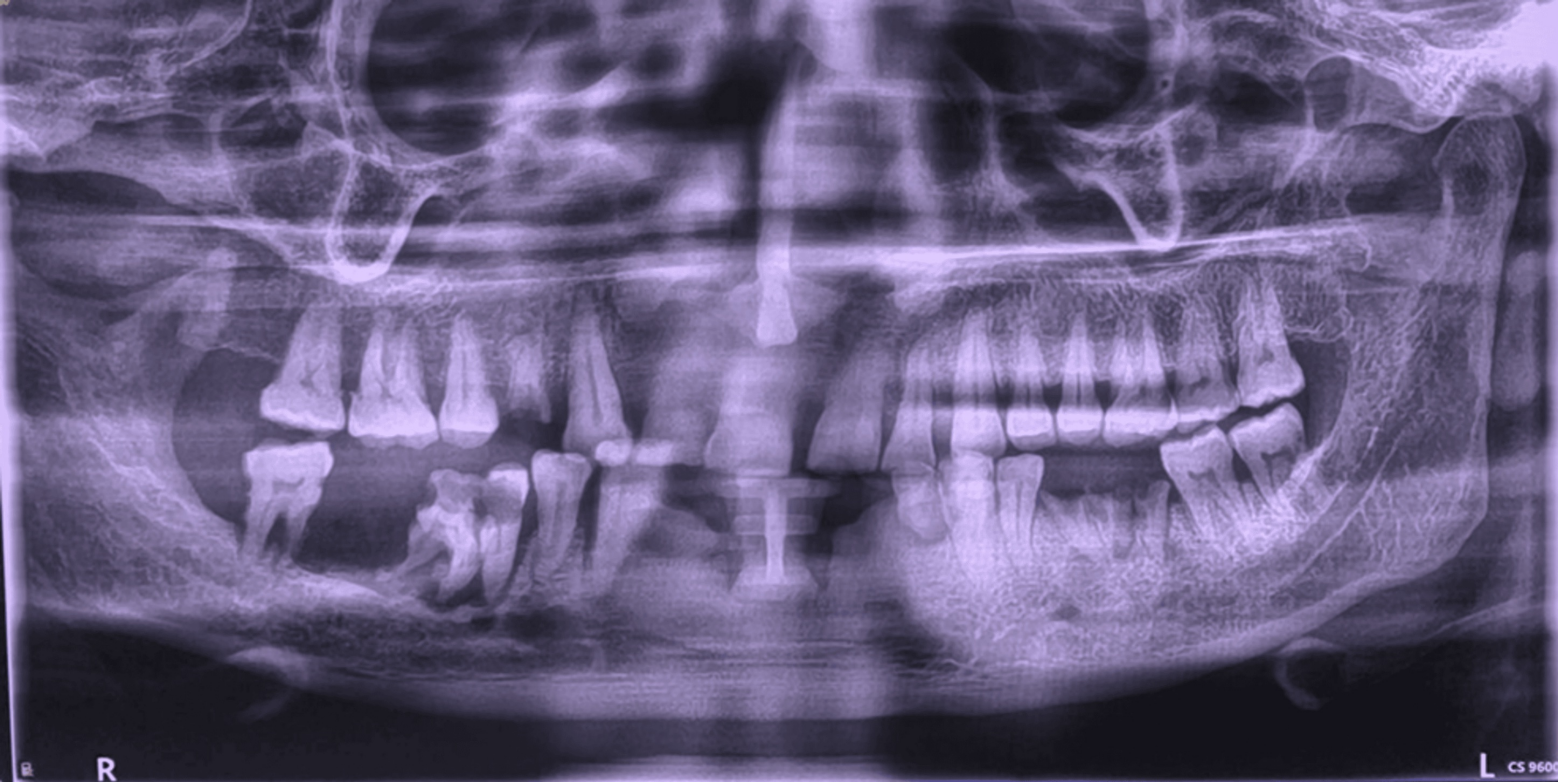
Orthopantomogram showing a well-defined radiolucent lesion in the posterior right mandibular region with associated tooth displacement and alveolar bone resorption

**Figure 3:**
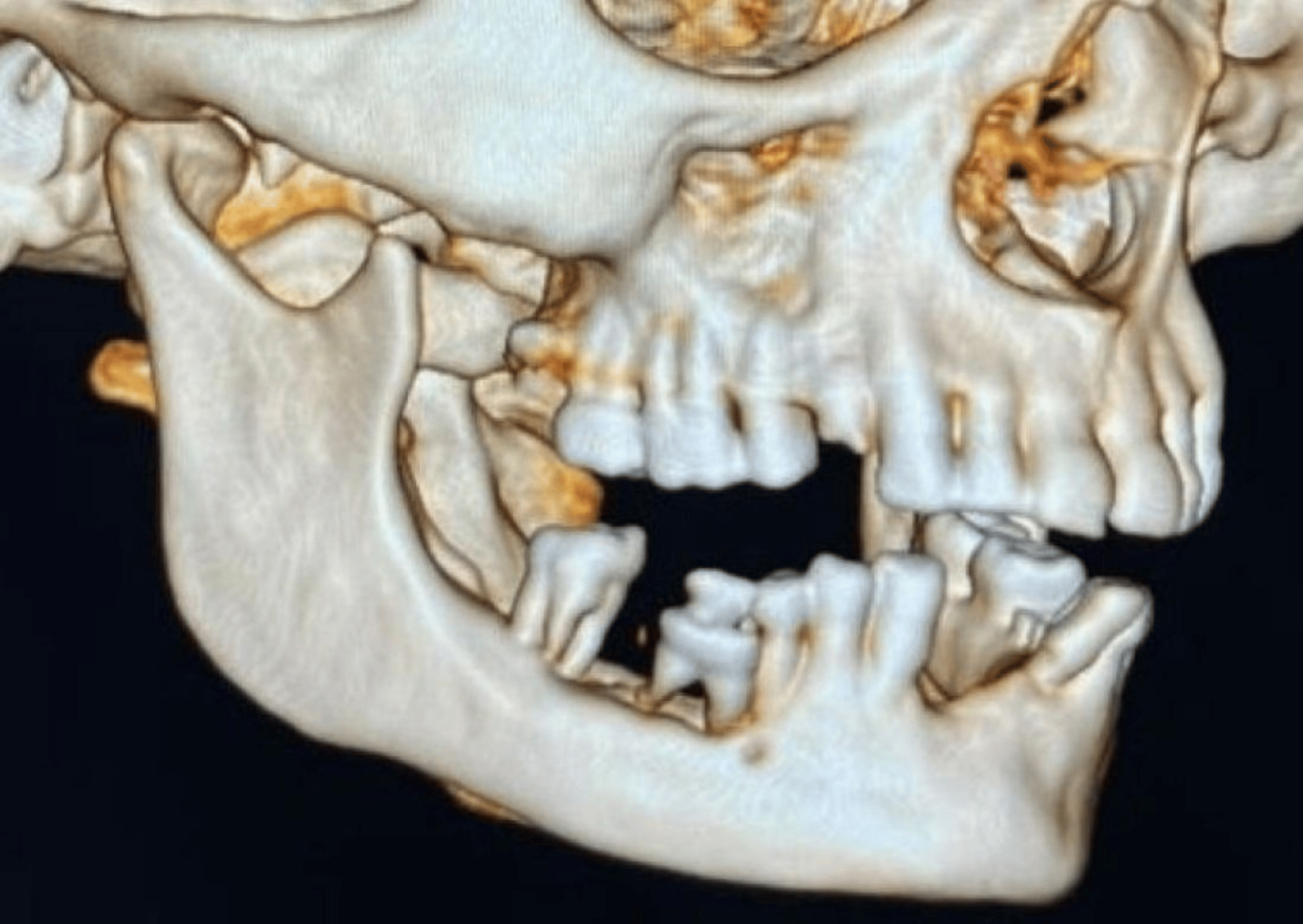
Contrast-enhanced CT scan revealing a heterogeneous lesion involving the posterior right inferior alveolus, measuring 2.4 × 2.7 × 2.1 cm

An orthopantomogram revealed a well-defined radiolucent area in the posterior right mandibular region, accompanied by displacement of adjacent teeth and localized alveolar bone loss (Figure 2). To further delineate the extent of the lesion, a contrast-enhanced CT scan was obtained. The CT scan demonstrated a heterogeneous soft-tissue mass with enhancing areas along the lingual aspect and centrally located hypo-enhancing regions suggestive of possible necrosis (Figure 3). The lesion measured approximately 2.4 × 2.7 × 2.1 cm and was closely associated with the molar roots. Lateral expansion into the right inferior gingivobuccal sulcus was evident; however, there was no cortical perforation, periosteal reaction, or extraosseous soft-tissue infiltration, and no extension into the retromolar trigone, floor of the mouth, or tongue musculature, helping to exclude malignant etiologies.

Given the clinical and radiological findings, a provisional diagnosis of PGCG was made; however, several differential diagnoses were considered. Central giant cell granuloma (CGCG) was considered due to its similar giant-cell histology, although it typically arises intraosseously rather than peripherally. Peripheral ossifying fibroma was considered because it presents as a firm gingival mass, but it usually contains calcifications, which are absent in PGCG. Pyogenic granuloma may resemble PGCG clinically but is more vascular and bleeds easily. A brown tumor of hyperparathyroidism was evaluated due to its giant-cell morphology, though it is distinguished by systemic biochemical abnormalities, which were not evident in this patient. Odontogenic tumors, such as ameloblastoma, can mimic bone involvement but are primarily intraosseous. Squamous cell carcinoma was also considered due to ulceration and bone loss, but its malignant epithelial features and invasive behavior differentiate it from PGCG.

**Figure 4:**
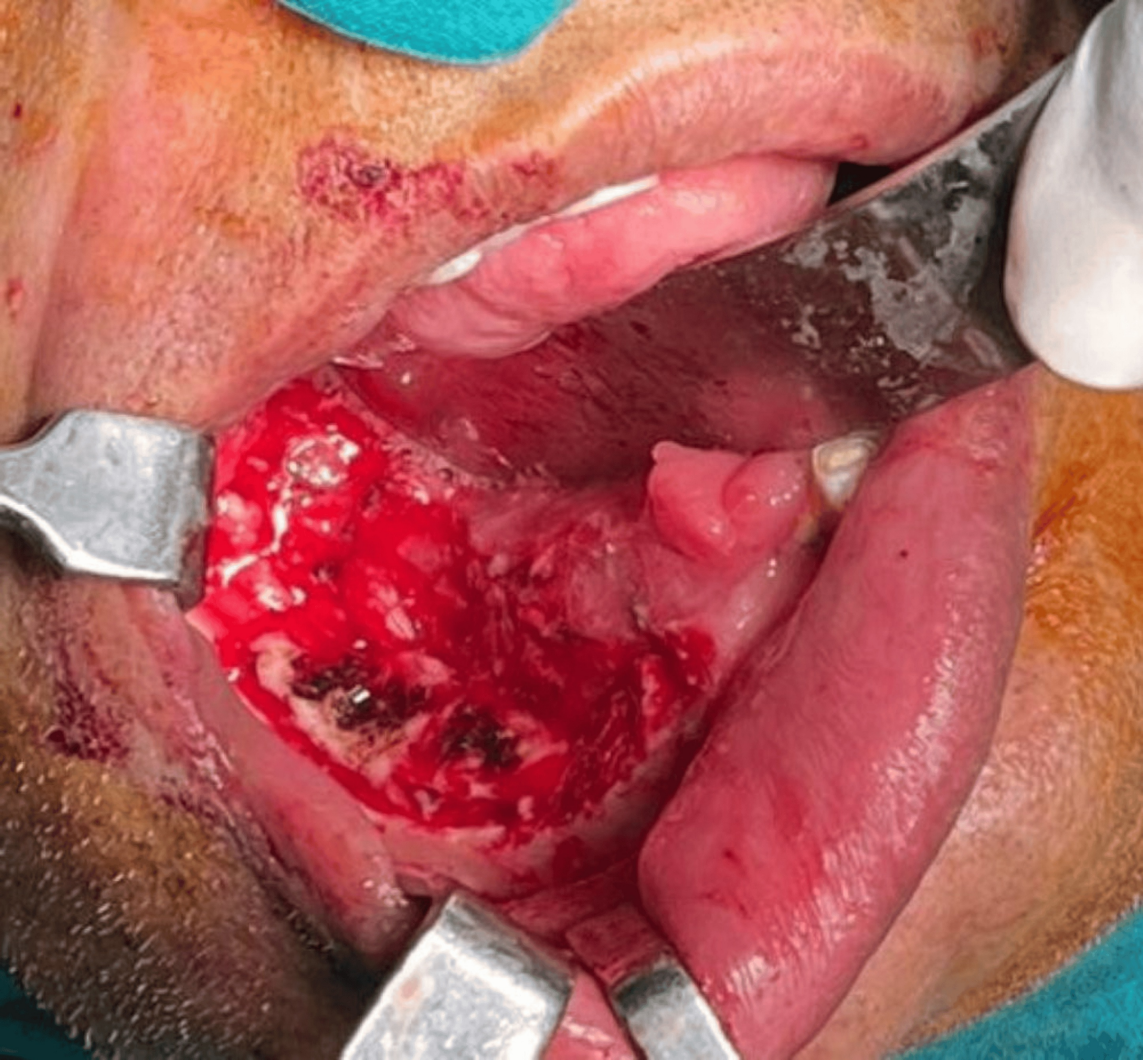
Intraoperative photograph showing surgical excision of the lesion with extraction of involved teeth and peripheral ostectomy performed under general anesthesia

The patient underwent surgical excision of the lesion under general anesthesia. Extraction of the compromised teeth (44, 45, 46, and 48) was performed, followed by peripheral ostectomy to remove residual tissue and minimize recurrence (Figure 4).

**Figure 5:**
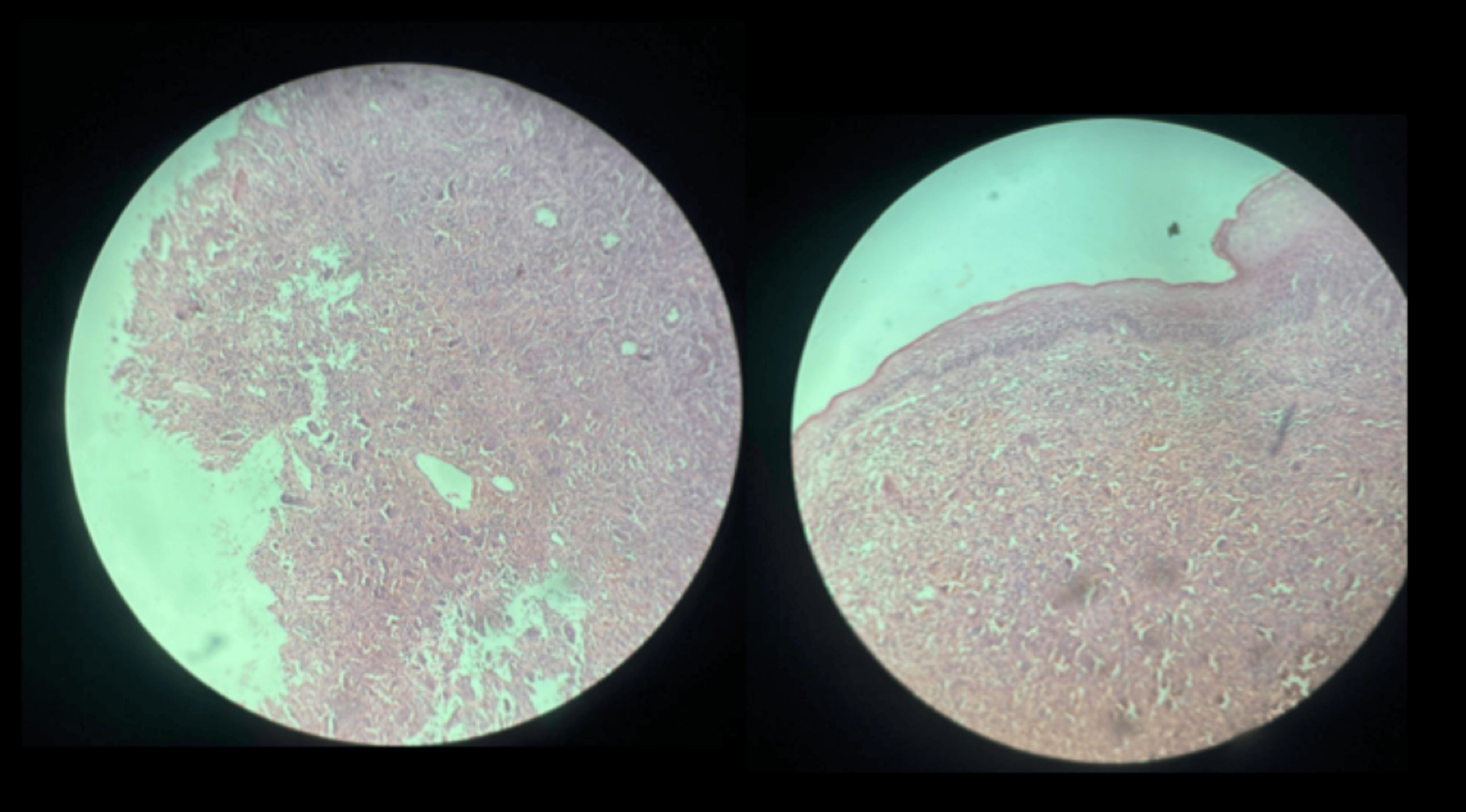
Hematoxylin and eosin-stained section showing hyperplastic stratified squamous epithelium with focal ulceration overlying inflamed granulation tissue The subepithelial connective tissue contains numerous multinucleated giant cells, mixed inflammatory infiltrate, and extravasated red blood cells.

Microscopic examination of the excised specimen revealed hyperplastic stratified squamous epithelium with focal ulceration overlying inflamed granulation tissue. The underlying connective tissue contained numerous multinucleated giant cells dispersed within an inflammatory, fibrovascular stroma. Red blood cell extravasation, hemosiderin-laden macrophages, and prominent vascularity were evident (Figure 5). No atypical mitotic figures, nuclear pleomorphism, or cellular atypia were observed on histopathological examination, supporting a benign reactive process. These findings confirmed the diagnosis of PGCG.

The postoperative course was uneventful. Follow-up examinations demonstrated satisfactory healing, with no evidence of recurrence at subsequent reviews.

## Discussion

PGCG is a relatively common, benign, reactive lesion of the oral cavity that originates from the periosteum or periodontal ligament in response to local irritation or trauma. It was first reported as “fungus flesh” in 1848. Historically, it has been referred to as giant cell reparative granuloma, osteoclastoma, giant cell epulis, and myeloid epulis, terms used interchangeably in older literature but no longer reflective of contemporary diagnostic distinctions. PGCG is a site-specific variant of pyogenic granuloma, containing osteoclast-like multinucleated giant cells and arising exclusively from the periodontal ligament surrounding the root of a tooth. It represents the soft tissue counterpart of CGCG, exhibiting similar histopathological features but a more limited clinical course. The lesion most frequently affects middle-aged individuals, with a slight female predilection, and is commonly located in the mandibular premolar-molar region [1,2].

Histologically, PGCG is characterized by an unencapsulated proliferation of multinucleated giant cells dispersed within a cellular, vascular connective tissue stroma containing hemorrhage, hemosiderin deposits, and chronic inflammatory cells [3,5]. The overlying stratified squamous epithelium may be ulcerated and replaced by a fibrinous exudate. Immunohistochemical and ultrastructural studies have demonstrated the presence of fibroblasts, endothelial cells, and osteoclast-like giant cells, supporting its reactive rather than neoplastic nature [5].

Clinically, the lesion presents as a sessile or pedunculated nodular mass with a reddish-blue hue due to its high vascularity. It may remain asymptomatic for long periods but can cause surface ulceration, bleeding, or displacement of adjacent teeth. Radiographically, a superficial “cupping” or saucer-shaped resorption of the underlying alveolar bone may be observed [1,6]. Although PGCG usually presents as a soft tissue lesion, its aggressive variants can mimic intraosseous lesions, warranting differential diagnosis from CGCG and other odontogenic or fibro-osseous pathologies [2,6].

According to Chrcanovic et al. and recent literature, the mandible remains the predominant site of occurrence, often without significant bone erosion; however, bone involvement has been reported in up to one-third of cases [7,8]. Therefore, appropriate investigations, including radiographic evaluation and serum assays for calcium, phosphate, alkaline phosphatase, and parathyroid hormone, are recommended to exclude systemic associations such as hyperparathyroidism.

Surgical excision remains the treatment of choice, performed down to the periosteum, along with removal of all local irritants such as calculus or defective restorations. The recurrence rate is reported to be between 10% and 16%, but adjunctive procedures such as curettage (2.8%) or peripheral ostectomy (0%) significantly reduce the likelihood of recurrence [7]. Other reported treatment modalities include sclerotherapy, marginal resection, and carbon dioxide laser excision, all of which have shown satisfactory healing with negligible differences in postoperative outcomes, though larger studies are needed for comparison [6]. Incomplete excision or failure to remove the periosteum has been correlated with higher recurrence rates [7].

Despite being extensively documented, PGCG still presents diagnostic challenges due to overlapping clinical and histological features with lesions such as pyogenic granuloma, peripheral ossifying fibroma, and even malignant entities. Limitations in the existing literature, such as retrospective study designs, inadequate follow-up, and lack of individualized recurrence data, warrant further long-term prospective studies to better define prognostic indicators and optimal management strategies [9].

## Conclusions

PGCG arising in the posterior mandible may exhibit an unusually aggressive radiographic appearance, leading to a clinical impression that mimics malignancy; however, histopathological evaluation can confirm the diagnosis of PGCG. Careful integration of clinical examination, radiographic imaging, and histopathological interpretation is essential for accurate diagnosis. Complete surgical excision, combined with the removal of potential local irritants, is crucial to preventing recurrence. Early recognition of atypical presentations ensures appropriate management, minimizes patient morbidity, and avoids misdiagnosis of more aggressive conditions. Consequently, regular follow-up and diligent oral hygiene are important, as these measures help reduce local irritants and may lower the risk of PGCG recurrence.
